# Impulsivity Moderates the Effect of Neurofeedback Training on the Contingent Negative Variation in Autism Spectrum Disorder

**DOI:** 10.3389/fnhum.2022.838080

**Published:** 2022-04-25

**Authors:** Karin Prillinger, Stefan T. Radev, Kamer Doganay, Luise Poustka, Lilian Konicar

**Affiliations:** ^1^Department of Child and Adolescent Psychiatry, Medical University of Vienna, Vienna, Austria; ^2^Department of Quantitative Research Methods, Institute of Psychology, Heidelberg University, Heidelberg, Germany; ^3^Department of Child and Adolescent Psychiatry and Psychotherapy, University Medical Center Göttingen, Göttingen, Germany

**Keywords:** executive functions, contingent negative variation, slow cortical potentials, neurofeedback, autism spectrum disorder, impulsivity, hierarchical models

## Abstract

**Background:**

The contingent negative variation (CNV) is a well-studied indicator of attention- and expectancy-related processes in the human brain. An abnormal CNV amplitude has been found in diverse neurodevelopmental psychiatric disorders. However, its role as a potential biomarker of successful clinical interventions in autism spectrum disorder (ASD) remains unclear.

**Methods:**

In this randomized controlled trial, we investigated how the CNV changes following an intensive neurofeedback training. Therefore, twenty-one adolescents with ASD underwent 24 sessions of slow cortical potential (SCP) neurofeedback training. Twenty additional adolescents with ASD formed a control group and received treatment as usual. CNV waveforms were obtained from a continuous performance test (CPT), which all adolescents performed before and after the corresponding 3-month long training period. In order to utilize all available neural time series, trial-based area under the curve values for all four electroencephalogram (EEG) channels were analyzed with a hierarchical Bayesian model. In addition, the model included impulsivity, inattention, and hyperactivity as potential moderators of change in CNV.

**Results:**

Our model implies that impulsivity moderates the effects of neurofeedback training on CNV depending on group. In the control group, the average CNV amplitude decreased or did not change after treatment as usual. In the experimental group, the CNV changed depending on the severity of comorbid impulsivity symptoms. The average CNV amplitude of participants with low impulsivity scores decreased markedly, whereas the average CNV amplitude of participants with high impulsivity increased.

**Conclusion:**

The degree of impulsivity seems to play a crucial role in the changeability of the CNV following an intensive neurofeedback training. Therefore, comorbid symptomatology should be recorded and analyzed in future EEG-based brain training interventions.

**Clinical Trial Registration:**

https://www.drks.de, identifier DRKS00012339.

## Introduction

The contingent negative variation (CNV) is an event-related potential (ERP) indicative of cognitive processes in continuous performance tasks (CPTs; Bekker et al., 2004). First described by Walter et al. (1964), the CNV is a slow, negative-polarity ERP occurring between a warning stimulus (S1) and an imperative stimulus (S2) (Tecce, 1972; Brunia and van Boxtel, 2001; Ahmadian et al., 2013). The CNV amplitude increases in anticipation of task performance and is thought to index changes in neural excitability in preparation for an approaching internal or external stimulus (Kononowicz and Penney, 2016). Furthermore, the CNV is associated with motor preparation and linked to response time (RT) and response time variability (Karalunas et al., 2014). The CNV is widely distributed over the scalp, with amplitudes typically peaking at frontal and central electrodes (Hamano et al., 1997). The primary neural generators of the CNV are thought to be located in the frontal cortex (Oishi and Mochizuki, 1998; Segalowitz and Davies, 2004; Battaglini et al., 2017), which plays a central role for exerting top-down response preparation and control (Rosahl and Knight, 1995; Brunia, 1999; Stuss and Alexander, 2007).

The CNV has been investigated mainly within the interpretative framework of executive functions (Flores et al., 2009). Executive functions represent higher-order cognitive processes that support goal-oriented behaviors, such as inhibitory control, problem solving, and cognitive flexibility (Cristofori et al., 2019). Imaging studies have demonstrated that these functions are represented by widespread brain networks, mainly localized in the pre-frontal cortex in conjunction with subcortical and allocortical regions, such as limbic areas and the hippocampus (Cristofori et al., 2019).

In typically developing (TD) participants, executive functions evolve at a different pace throughout development and continue to mature in adolescence (Best and Miller, 2010). Despite some older studies reporting an increasing CNV in pre-adolescence with maximum amplitude at 15 years (Tecce, 1971, 1972), more recent studies suggest that the CNV amplitude increases linearly from childhood through adolescence and adulthood, in line with the assumed maturation of executive function abilities in general (Segalowitz and Davies, 2004; Jonkman, 2006; Segalowitz et al., 2010). In TD children and adults, a larger CNV amplitude is associated with stronger response preparation and better performance in executive function tasks (Segalowitz et al., 1992; Dywan et al., 1994; Jonkman, 2006).

Another interesting research avenue has sought to unravel group differences between TD controls and psychiatric patients regarding the CNV as an indicator of executive processes. For instance, in several neuropsychiatric disorders, a lower CNV amplitude was found compared to TD controls, such as patients with schizophrenia and bipolar disorder (Li et al., 2015), patients with schizophrenia (Wang et al., 2020) and patients suffering from seizures (Drake et al., 1997). The CNV has also been investigated as a neurophysiological biomarker in functional movement disorders (Teodoro et al., 2020), psychomotor dysfunction in schizophrenia (Juston Osborne et al., 2020) and post-traumatic stress disorder after an acute trauma (Kowalski et al., 2018). Moreover, the CNV has also been utilized to asses changes in the dopaminergic function induced by pharmacological treatments (Linssen et al., 2011). However, besides a study on the CNV as a treatment response to an intervention targeting abnormal attention in functional movement disorders (Teodoro et al., 2020), almost all studies investigating CNV changes as an outcome measure were conducted as part of ADHD neurofeedback research (for an overview see: Mayer et al., 2013). These intervention studies showed increases in CNV amplitudes and reduced ADHD symptomatology after neurofeedback training (Mayer et al., 2013, 2016; Gevensleben et al., 2014).

Another common psychiatric disorder associated with an array of executive dysfunctions is Autism Spectrum Disorder (Demetriou et al., 2018). Autism Spectrum Disorder (ASD) is a neurodevelopmental disorder defined by social and communicative deficits and repetitive, stereotyped behaviors (International Classification of Diseases ICD-10; WHO, 2016). Impaired executive functions in ASD have been suggested as a model for understanding behavioral problems associated with the disorder (Demetriou et al., 2018, 2019). Furthermore, common impairments in the executive function abilities such as set shifting, response inhibition, and working memory negatively impact indicators of mental health, social interaction, and lifelong functioning outcomes in ASD (Demetriou et al., 2019).

Neuroimaging studies have linked difficulties in executive functions to altered functional connectivity in ASD (Kana et al., 2007; Koshino et al., 2008; Braden et al., 2017). A recent meta-analysis reported ASD-related activation abnormalities in the left inferior frontal gyrus, left inferior parietal gyrus and sensorimotor areas during the performance of executive function tasks (Zhang et al., 2020). The results suggest that the mechanisms underlying executive function difficulties in ASD are relatively stable across all age groups, which is in line with behavioral data suggesting executive dysfunctions throughout development (Lai et al., 2017; Zhang et al., 2020). However, despite measurable executive dysfunctions, adults with ASD show a slightly better performance on some executive function tasks due to improved compensatory strategies and/or developmental maturity (Demetriou et al., 2018). Thus, investigating the CNV in ASD populations might provide valuable information beyond behavioral data about the underlying neurocognitive sources of executive difficulties.

However, studies analyzing the CNV in participants with ASD have yielded mixed results (Strandburg et al., 1993; Tye et al., 2014; Thillay et al., 2016; Høyland et al., 2017; Hoofs et al., 2018). Early studies could not find any differences between CPT performance and CNV amplitude of high-functioning adults with ASD compared to TD adults (Strandburg et al., 1993). This result was partly supported by a study on adolescents with ASD, showing no differences between adolescents with ASD compared to TD controls aged between 12 and 15 years, whereas for older adolescents (older than 16 years), the CNV was found to be enhanced (Høyland et al., 2017). Similarly, Tye et al. (2014) reported increased CNV amplitudes in participants with ASD compared to participants with ADHD, ASD + ADHD, or TD controls. No differences in the behavioral outcomes of the CPT were registered between children with ASD and TD children (Tye et al., 2014). Thus, comorbidity factors seem to affect the expression of CNV in ASD and need to be taken into account in future analyses. In addition, Thillay et al. (2016) found an increased CNV amplitude comparing adults with ASD and TD adults. Only one study reported a decreased CNV in adolescents and young adults with ASD compared to a TD control group (Hoofs et al., 2018). In this study, the ASD group did not show CNV differentiation based on the task specifics, indicating that ASD participants may have deficits in fine-tuning their intentional control to negligible conditions (Hoofs et al., 2018). This result further points toward an altered top-down response preparation in ASD (Høyland et al., 2017).

With this study, we aim to quantify the effects of neurofeedback training on the CNV in children and adolescents diagnosed with ASD. Given the mixed and scant results obtained in the context of ASD and CNV, we refrained from formulating a directional hypothesis and performed our analyses in an exploratory manner. Furthermore, as indicated in previous studies, we explore the role of three components of comorbid ADHD symptoms, namely, inattention, hyperactivity, and impulsivity, as potential moderators. In addition, we consider the age of participants as a potential control variable, as suggested by the literature. In order to utilize the entire data available, we fit a multivariate hierarchical Bayesian model to trial-level CNV amplitudes obtained prior to and following neurofeedback training. Finally, we elucidate the importance of using the finer-grained separation of comorbid ADHD symptoms instead of aggregate comorbid scores.

## Materials and Methods

### Experimental Design

**FIGURE 1:**
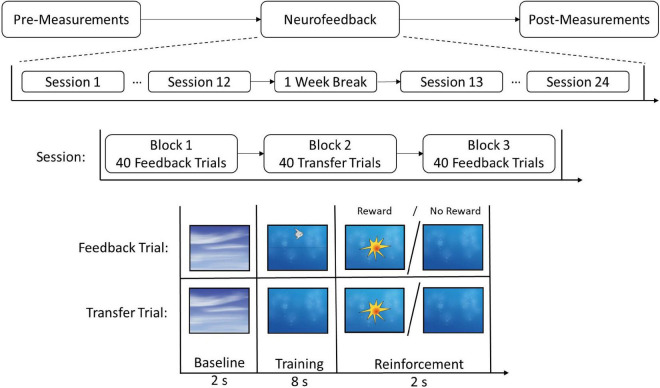
Experimental timeline for the neurofeedback group. Participants received 24 sessions of neurofeedback, including a 1 week break between sessions 12 and 13. Every session entailed three blocks and each block consisted of either 40 Feedback or 40 Transfer trials. The trials included a baseline, a training, and a reinforcement phase. In the reinforcement phase, participants received positive visual feedback (reward) if the trial was successful and no feedback if the trial was not successful. Copyright Neurofeedback Screens © neuroConn GmbH/neurocare group AG.

The present report is part of a randomized, controlled clinical intervention trial (Clinical Trial Registration: DRKS00012339) using the same sample of adolescents with ASD as in Konicar et al. (2021). This study investigated whether the core symptomatology of ASD could be reduced via an electroencephalogram (EEG)-based brain self-regulation training of SCPs. For this reason, study participants with ASD were randomly assigned to an experimental group (EG) (*N*_1_ = 21) or a control group (CG) (*N*_2_ = 20). In the EG, each adolescent participated in a series of 24 neurofeedback training sessions. Training sessions took place twice a week. Each neurofeedback session consisted of three trainings blocks, each lasting 8 min, with a total of 120 trials. During the two feedback blocks, participants received information about their ongoing EEG activity. The middle block was a transfer block where no feedback was provided (see Figure 1). During the feedback trials, SCPs recorded from the EEG channel FCz were presented on the participants screens via a moving graphical object (e.g., a fish). The participants in the CG received treatment as usual (TAU, e.g., counseling; for further details, see Konicar et al., 2021). In the week prior to the first neurofeedback or TAU intervention and the week after the 3 months’ intervention phase, CNV was recorded during the administration of the CPT. Also, parental ratings regarding comorbid ADHD symptoms were obtained using the Diagnostic System for Mental Disorders in Childhood and Adolescence (DISYPS II; Döpfner et al., 2008), which comprises an ADHD total score and subscales for inattention, hyperactivity, and impulsivity.

### Participants

Data from forty-two right-handed, male adolescents with a diagnosis of ASD were analyzed for this study. One participant was excluded from analysis due to incomplete data (final sample *N* = 41, range 12–17 years, mean age ± standard deviation 14.05 ± 1.76 years). Inclusion criteria comprised ASD diagnostics based on ADI-R (Diagnostic Interview for Autism-Revised, German version; Bölte et al., 2006) and ADOS-2 (Diagnostic Observation Schedule for Autistic Disorders, German version; Poustka et al., 2015). Exclusion criteria were an IQ lower than 70 and medical conditions that could impair the neurofeedback training or physiological measures (head injuries, major Axis I diagnosis of psychosis, obsessive-compulsive disorder, severe motor or vocal tics, Tourette syndrome, or severe depression with suicidality). All participants had normal or corrected-to-normal vision and no prior experience with neurofeedback. Pharmacological and psychosocial interventions were allowed if they were kept constant during study participation. The study was approved by the Ethics Committee of the Medical University of Vienna and conducted in line with the Declaration of Helsinki.

### Continuous Performance Task (CPT-OX): Electrophysiological and Behavioral Measures

The cued Continuous Performance Task (CPT-OX, neuroConn GmbH, Ilmenau, Germany) comprising 400 arrays of 100 target black letters (4 sequences of 100 letters each, with 11 different letters; H, J, K, O, X, D, F, G, M, L, D), was presented over a white background at the center of a computer screen. The letters were presented for 150 ms every 1650 ms in a pseudo-randomized order at the center of the monitor. Participants were instructed to only press a dedicated joystick button when a letter “X” followed a letter “O” (Go trial). From the 400 trials, the letter “O” was presented 80 times (Cue trial), followed 40 times by an “X” (Go trial) and 40 times by an irrelevant letter (No-go trial). Participants were instructed in a written and verbal fashion about the structure of the task and told to answer as fast and as accurate as possible. The task was practiced and comprehension was verified prior to the start of the first measurement (pre). The duration of the task was about 10 min.

For the behavioral task, hits (correct button press to target stimuli), omission errors (no button press, where a button press was required), commission errors (false button press), as well as mean reaction times (RT) and standard deviation (SD) were recorded by the Theraprax EEG System (neuroConn GmbH, Ilmenau, Germany).

Electrophysiological data was recorded via Ag/AgCl electrodes from four centro-medial EEG channels (Fz, FCz, Cz, and Pz) according to the 10–20 system using the Theraprax EEG System (neuroConn GmbH, Ilmenau, Germany). Recordings were conducted with a 128 Hz sampling rate and the reference electrode positioned at the right mastoid. Horizontal and vertical electro-oculography (EOG) were recorded with four electrodes, below and above the right eye and at the outer canthi of both eyes.

### EEG Data Preprocessing

**FIGURE 2:**
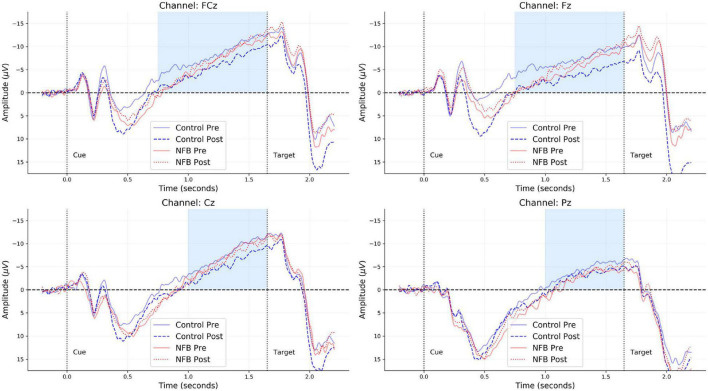
Grand-averaged CNV waveforms for each of the four channels (FCz, Fz, Cz, and Pz) obtained from each group (neurofeedback vs. control) and time point (pre vs. post). The light blue regions indicate the time-window considered for computing the mean CNV amplitude within each trial of the CPT.

All offline EEG preprocessing steps were performed using MNE-Python (Gramfort et al., 2014) and the *R* programming language. A zero-phase finite impulse response (FIR) filter with bandpass frequency 0.01–30 Hz was used to filter the raw EEG data. The filtered data for each participant in each *Group* (neurofeedback vs. control) and at each *Time* (pre vs. post) were segmented into epochs comprising a 0.2-s baseline window and a 2-s trial window. Epochs were then baseline corrected by subtracting the mean amplitude in the baseline window from each corresponding trial window. Rejection of artifactual trials was performed in a two-step manner. As a first step, trials with a peak-to-peak amplitude exceeding 150 μ*V* or exhibiting marked ocular activity were flagged as bad and removed from subsequent analyses^1^. Epochs which remained after this step were reduced to a single numeric summary by computing the mean EEG amplitude between 0.75 and 1.65 s of the trial window, defined as the CNV window. As a second step, a Hampel filter was used to remove all averaged epochs with median absolute deviation (MAD) above or below five. Overall, for each group and time point, more than 80% of all trials remained [Range: (84–86%)] after this step. No further data aggregation was performed, since the trial-averaged amplitudes were used as a basic unit for our multilevel analysis. Grand-average CNV waveforms for all four electrodes are depicted in Figure 2 merely for visual inspection and alignment with previous CNV studies.

### Bayesian Multilevel Analysis of Trial-Level Data

For the primary analyses, we fit the following hierarchical model, which represents the average CNV amplitude obtained from each trial as a linear function of the following regressors:


T⁢r⁢i⁢a⁢l⁢A⁢m⁢p⁢l⁢i⁢t⁢u⁢d⁢e=G⁢r⁢o⁢u⁢p*T⁢i⁢m⁢e*A⁢D⁢H⁢D+A⁢g⁢e+(1|I⁢D)

This binary moderator model assumes an interaction between the categorical factors *Group* and *Time* and outsources participant variability to a *random intercept* component for each participant. The *ADHD* score represents an aggregate binary clinical score computed from the DISYPS II (Döpfner et al., 2008) subscale scores *Impulsivity*, *Attention*, and H*yperactivity* (total stanine score >7). We treat the binary clinical scores both as a main effect and as a moderator of the interaction between *Group* and *Time.* Differently, we include *Age* as a standalone predictor (i.e., main effect), and treat it as a control variable. We fit a multivariate hierarchical model to account for CNV amplitudes recorded at all four channels while simultaneously considering residual correlations between the channels.

In a second step, in order to obtain a fine-grained picture of the moderating effect of ADHD comorbidity, we fit the following extended model, which represents the average CNV amplitude in each trial as a linear function of the following regressors:


T⁢r⁢i⁢a⁢l⁢A⁢m⁢p⁢l⁢i⁢t⁢u⁢d⁢e=G⁢r⁢o⁢u⁢p*T⁢i⁢m⁢e*(I⁢m⁢p⁢u⁢s⁢i⁢v⁢i⁢t⁢y+A⁢t⁢t⁢e⁢n⁢t⁢i⁢o⁢n+H⁢y⁢p⁢e⁢r⁢a⁢c⁢t⁢i⁢v⁢i⁢t⁢y)+A⁢g⁢e+(1|I⁢D)

T⁢r⁢i⁢a⁢l⁢A⁢m⁢p⁢l⁢i⁢t⁢u⁢d⁢e

=

Group*Time*(Impusivity+Attention

+Hyperactivity)+Age+(1|ID)

This fine-grained moderator model also assumes an interaction between the categorical factors *Group* and *Time* and outsources participant variability to a *random intercept* component for each participant. However, instead of a binary aggregate variable, we include the DISYPS II (Döpfner et al., 2008) subscales scores *Impulsivity*, *Attention*, and *Hyperactivity* as control variables and as moderators of the interaction between *Group* and *Time.* Once again, we consider *Age* as a standalone predictor (i.e., a control variable) and fit a multivariate model to account for CNV amplitudes measured at all four channels. Importantly, this model does not explicitly represent dependencies between the three subscales, as the latter would disproportionally increase the complexity and interpretability of the model.

Both models were estimated using the *R*-package *brms f*or fitting and comparing Bayesian multilevel models (Bürkner, 2017). We assumed broad priors for all intercept and weight parameters, that is, Normal(0, 50), and used default priors for the variance parameters. We confirmed convergence of the Markov chains for each model parameter via visual inspection of the chains and inspection of the Gelman-Rubin convergence metric $\hat{R}$. We also performed posterior model checking for ascertaining reasonable recovery of individual and population-level mean CNV amplitudes.

## Results

### Behavioral Data (CPT-OX)

We found no behavioral differences in reaction time, standard deviation of reaction times, hits, omission errors or commission errors between the groups or time points (see Table 1).

**TABLE 1:** Descriptive data for behavioral measures.

	EG		CG	
	Pre (*n* = 21)	Post (*n* = 20)	Pre (*n* = 20)	Post (*n* = 20)
Hits	38.38	38.4	37.40	37.4
Omission errors	1.62	1.6	2.60	2.6
Commission error	0.76	1.05	0.75	0.35
RT	383	386	355	357
RT SD	152	149	123	119

*RT, mean reaction time in milliseconds (ms); RT SD, standard deviation of the mean reaction time in ms; behavioral data of one participant in the EG had to be excluded at post due to technical issues during the recording.*

### Contingent Negative Variation Analyses: Binary Moderator Model

**FIGURE 3:**
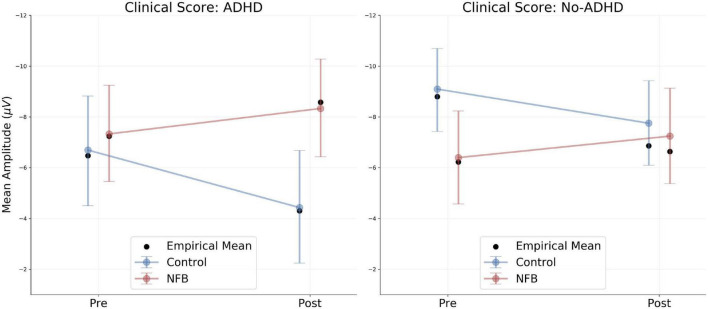
Binary moderator model results. Model-based predictions of population-level CNV amplitudes across both levels of the binary moderator. Colored points indicate predicted means. Red, Neurofeedback Group (NFB); Blue, Control Group. Black points indicate empirical means. Notice the difference between predicted and observed means due to shrinkage. Error bars indicate 95% credibility intervals (predictive uncertainty).

A summary of the resulting model parameter estimates is presented in Table 2. Overall, we observe large uncertainty around the parameter estimates due to the high variability of trial-based CNV amplitudes. In order to ease interpretability, we derive model-based predictions for the population-level means across both levels of the binary moderator (see Figure 3).

**TABLE 2:** Summary of the results obtained by the binary moderator model of CNV amplitude at channel FCz.

Predictor	Estimate	Standard error	95%–CI
**Population-level effects**			
(Intercept)	−4.69	3.47	(−11.48 to 2.1)
Time:Post	2.26	1.39	(−0.48 to 4.95)
Group:NFB	−0.66	1.46	(−3.53 to 2.19)
No-ADHD	−2.41	1.38	(−5.07 to 0.34)
Age	−0.14	0.23	(−0.6 to 0.31)
Time:Post × Group:NFB	−3.26	1.84	(−6.83 to 0.33)
Time:Post × No-ADHD	−0.89	1.75	(−4.32 to 2.52)
Group:NFB × No-ADHD	3.37	1.98	(−0.53 to 7.2)
Time:Post × Group:NFB × No-ADHD	1.05	2.39	(−3.68 to 5.75)
**Family-specific and group-level effects**			
Trial-Variability (σ)	21.89	0.21	(21.48 to 22.32)
Person-Intercept-Variability (τ_00_)	1.21	0.39	(0.42 to 2.00)

*Parameter means, standard deviations, and 95% credibility intervals (CI) are reported as summaries of the respective posterior distributions.*

Notably, the clinical Total ADHD Score (DISYPS II; Döpfner et al., 2008) does not seem to have a different influence on the change in CNV amplitude within each group: average CNV amplitude increases in the neurofeedback (NF) group and decreases in the control group (CG) from pre to post intervention. However, the starting points of each group are radically different across the two ADHD categories. Participants in both groups scoring above the cut-off for ADHD exhibit similar CNV amplitudes prior to the intervention. Following the intervention, the two groups diverge with respect to average CNV amplitudes (cf. Figure 3, left panel). Differently, participants scoring below the cut-off for ADHD exhibit widely different CNV amplitudes prior to the intervention, depending on which group they were assigned to. Following the intervention, mean CNV amplitudes in both groups appear to converge (cf. Figure 3, right panel).

### Contingent Negative Variation Analyses: Fine-Grained Moderator Model

**FIGURE 4:**
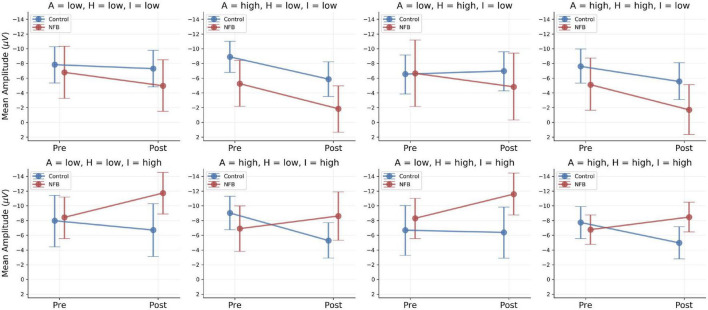
Fine-grained moderator model results. Model-based predictions of population-level means across low and high scores of the moderator variables: A, Attention; H, Hyperactivity; I, Impulsivity. Points indicate predicted means: Red, Neurofeedback Group (NFB); Blue, Control Group. Error bars indicate 95% credibility intervals (predictive uncertainty). Only model-based predictions are depicted, since quasi-continuous moderators are conditioned on concrete values and no discrete subgroups were built for analysis.

A summary of the resulting model parameter estimates is presented in Table 3. Once again, we observe large uncertainty around the estimated regression parameters due to the high variability of trial-based CNV amplitude. Notably, in this model, the estimated main effect of age, β=−0.62, (95%-CI [−1.1 to −0.12], β = 0 not included in the 95% CI), indicates an increase in CNV amplitude with increasing age. In addition, the moderating effect of *Impulsivity* on the interaction between *Time* and *Group*, β=−1.95, (95%-CI [−3.69 to −0.21], β = 0 not included in the 95% CI), suggests a noticeable increase in average CNV amplitude for participants in the NF group scoring high on the impulsivity subscale (DISYPS II; Döpfner et al., 2008; see Figure 4, second row).

**TABLE 3:** Summary of the results obtained by the fine-grained moderator model of CNV amplitude at channel FCz.

Predictor	Estimate	Standard error	95%–CI
**Population-level effects**			
(Intercept)	1.15	5.58	(−9.81 to 12.11)
Time:Post	−3.90	5.31	(−14.22 to 6.53)
Group:NFB	0.67	7.16	(−13.41 to 14.53)
Attention	−0.35	0.64	(−1.6 to 0.88)
Hyperactivity	0.43	0.24	(−0.05 to 0.9)
Impulsivity	−0.05	0.30	(−0.63 to 0.54)
Age	−0.62	0.25	(−1.1 to −0.13)
Time:Post × Group:NFB	12.17	8.76	(−4.84 to 29.59)
Time:Post × Attention	0.83	0.81	(−0.75 to 2.41)
Time:Post × Hyperactivity	−0.32	0.31	(−0.94 to 0.29)
Time:Post × Impulsivity	0.24	0.39	(−0.51 to 1.01)
Group:NFB × Attention	0.86	0.82	(−0.73 to 2.49)
Group:NFB × Hyperactivity	−0.38	0.49	(−1.34 to 0.59)
Group:NFB × Impulsivity	−0.49	0.71	(−1.85 to 0.89)
Time:Post × Group:NFB × Attention	−0.29	1.03	(−2.28 to 1.71)
Time:Post × Group:NFB × Hyperactivity	0.32	0.65	(−0.94 to 1.58)
Time:Post × Group:NFB × Impulsivity	−1.95	0.90	(−3.69 to −0.21)
**Family-specific and group-level effects**			
Trial-Variability (σ)	21.88	0.22	(21.46 to 22.30)
Person-Intercept-Variability (τ_00_)	1.14	0.45	(0.21 to 2.06)

*Parameter means, standard deviations, and 95% credibility intervals (CI) are reported as summaries of the respective posterior distributions.*

In order to probe the effects of the three moderator variables [subscales *Attention (A), Hyperactivity (H), Impulsivity (I)*], we determined low and high scores on the three ADHD scales directly from the sample distribution by computing *median* ± MAD for each subscale score. Accordingly, low scores for *Impulsivity*, *Attention*, and *Hyperactivity* were defined at the values 5.5, 4.5, and 5.5 and high scores at the values 8.5, 7.5, and 8.5, respectively. We then investigated the group-level conditional predictions of the model for each of the eight combinations arising by considering the low and high scores of the three moderators. These results are depicted in Figure 4.

## Discussion

In this work, we investigated changes in the CNV following an intensive neurofeedback training for adolescents with ASD and comorbid ADHD symptoms. In order to obtain a more nuanced exploratory analysis, we formulated and fitted two hierarchical moderator models: one model containing a binary aggregate ADHD score as a single moderator and one model containing three separate moderators representing quasi-continuous scores for inattention, hyperactivity, and impulsivity. Our main goals were to show that (*i*) utilizing the non-aggregated trial-based EEG data can yield meaningful predictions and uncertainty intervals; (*ii*) comorbid ADHD symptoms comprise an important factor to consider when investigating the CNV in ASD populations and (*iii*) disentangling binary ADHD scores into their underlying sub-components can provide more information on how comorbidity in ASD affects changes in CNV.

Accordingly, our binary moderator model reveals no influence of the aggregate ADHD score on the observed CNV trajectories (slopes) across the two time points. However, differentiating between adolescents with comorbid ADHD and adolescents without comorbid ADHD, the model predicts radically different CNV amplitudes between the control and the neurofeedback group at baseline. Participants with ASD and comorbid ADHD exhibit similar CNV amplitudes prior to the intervention, irrespective of their group. After the intervention, the average CNV amplitude in the neurofeedback group *increases*, whereas the average CNV amplitude in the control group *decreases*, resulting in a diverging pattern (cf. Figure 3, left panel). Differently, participants without comorbid ADHD exhibit widely different CNV amplitudes prior to the intervention: The average CNV amplitude in the control group is much higher than the average CNV amplitude in the neurofeedback group. Following the intervention, the average CNV amplitude in the neurofeedback group *increases*, whereas the average CNV amplitude in the control group *decreases*, resulting in a converging pattern (cf. Figure 3, right panel). The results obtained for participants without comorbid ADHD are difficult to interpret due to the differences in baseline CNV, since treatment-induced change might easily be confounded with regression to the mean. Larger samples or hypothesis-driven stratification for small samples might overcome unbalanced baseline distributions in future studies.

The fine-grained moderator model implies a rather complex pattern. In the control group, the CNV amplitude either decreased or remained relatively stable across the eight symptom-severity combinations. In the experimental group, two different patterns emerged depending on the severity of comorbid impulsivity symptoms. Whereas participants with low impulsivity scores showed a decreased CNV amplitude, participants with high impulsivity scores exhibited an enhanced CNV amplitude after neurofeedback intervention (cf. Figure 4, second row). The other two comorbid symptoms – inattention and hyperactivity – did not seem to exert a noticeable influence on the group-dependent change in CNV amplitude. This suggests that impulsivity might be the primary ADHD component driving changes in CNV. Considering the current results, future studies investigating the response of the CNV to different clinical interventions in ASD should include a measure of impulsivity as a moderating factor.

Furthermore, age appears to be an important predictor of CNV in the fine-grained model, implying that the average CNV amplitude increases with participant’s age. Considering that the CNV changes with age in TD persons (Jonkman, 2006), this result is line with previous research. However, results regarding CNV amplitudes and developmental trajectories in ASD are mixed (Strandburg et al., 1993; Tye et al., 2014; Thillay et al., 2016; Høyland et al., 2017) and the current study only provides a cross-sectional view on the effects of age.

Although impulsivity as a construct is generally well investigated, a comprehensive theory which overcomes major challenges of theoretical specifications, multiplicity of definitions, and measurement approaches, is still missing (Strickland and Johnson, 2021). Moeller et al. (2001) described impulsivity as a biopsychosocial construct encompassing a reduced sensitivity to negative consequences of behavior, rash responses to stimuli without thoroughly processing the available information, and little to no regard for long-term consequences of behavior regarding all actors involved in a situation. Further characteristics of impulsivity are a lack of inhibition and reflection in decision making, deficits in delaying gratification, premature behavior and inability to resist impulses and urges (Robbins et al., 2012; Berlin and Hollander, 2014). Impulsive behavior is commonly described in diverse psychiatric disorders, such as ADHD, addiction, or bipolar disorder as well as in antisocial and aggressive disorders (Moeller et al., 2001; Robbins et al., 2012). Furthermore, impulsive behaviors are considered as a risk factor for the development of substance abuse and addiction (Verdejo-García et al., 2008; De Wit, 2009; Robbins et al., 2012) and facilitate non-suicidal self-injury behaviors and suicide attempts (Corruble et al., 1999).

Frontal brain areas have been reported as common neural substrate for impulsive behaviors (Berlin and Hollander, 2014). In particular, fronto-striatal circuitries, stemming from striatal projections, have been associated with impulsive behavior and deficits in inhibitory prefrontal control (Dalley et al., 2011; Berlin and Hollander, 2014). Also, studies have found an association between the generation and amplitude of the CNV and the activation of a thalamo-cortico-striatal network (Ikeda et al., 1997; Nagai et al., 2004; Fan et al., 2007). BOLD activity has been associated with the trial-by-trial variation in CNV amplitude in the bilateral thalamus, the anterior cingulate, and the supplementary motor cortex (Nagai et al., 2004). Strong relationships between the CNV and impulsivity have also been reported in offenders with antisocial disorder, for whom the CNV have already been suggested as a potential predictor of recidivism (Howard, 2002). Therefore, impulsivity has been assumed to arise as a result of impaired response inhibition, that is, a deficient top-down control (Dalley et al., 2011).

Neurofeedback training has been suggested as a viable treatment for impulsivity and inattention in children with ADHD (Arns et al., 2009). However, in our fine-grained moderator model, inattention seemed to exert little influence on CNV patterns and we could not find intervention-specific behavioral improvements in the CPT regarding sustained attention, which is in line with CPT results by Pamplona et al. (2020). Nevertheless, using network-based real-time functional magnetic resonance imaging, Pamplona et al. (2020) found improved temporal sustained attention following neurofeedback training. Thus, initial results underline the potential of neurofeedback as a non-pharmacological method to enhance attention and possibly other cognitive functions in psychiatric disorders.

Regarding further neurophysiological changes, increased CNV amplitudes have been found in patients with ADHD after neurofeedback training (see Mayer et al., 2013). However, to our knowledge, no other study explored the effects of subcomponents of ADHD symptomatology on CNV changes. Importantly, ADHD is a common comorbidity of ASD, with co-occurrence rates ranging from 37% to 85% (Leitner, 2014; Stevens et al., 2016; Gnanavel et al., 2019). Both disorders have been associated with abnormal allocation of attentional resources and performance monitoring (Lau-Zhu et al., 2019).

A consideration of ADHD and ASD from a transdiagnostic perspective based on the Research Domain Criteria Initiative identifies three distinct transdiagnostic subtypes of executive function profiles. Subtypes are defined by deficits in (1) behavioral flexibility and emotion regulation; (2) hyperactivity/impulsivity and inhibition; and (3) working memory, organizing, and planning (Vaidya et al., 2020). Alongside this subtype focus, our findings contribute to the investigation of potential alternative or adjunct treatment approaches for ASD, revealing fine-grained differences in expected treatment outcomes depending on the comorbid subtypes or even a transdiagnostic psychopathological construct.

### Limitations

During this study, participants were allowed to continue their psychopharmacological treatments. However, a study in TD adults investigated the effects of different doses of methylphenidate on the CNV and found a dose-dependent increase in CNV amplitude indicative of a heightened response readiness (Linssen et al., 2011). Moreover, changes in the CNV amplitude could be detected even after a dose as low as 10 mg, demonstrating the sensitivity of the CNV (Linssen et al., 2011). Since the relationship between methylphenidate, SCP neurofeedback training, and the CNV is unclear, a confounding effect of the psychopharmacological medication on the results of this study are possible.

Further, given the resolution of our model description, we could not ascertain the precise causal mechanisms underlying the statistical interactions implied by our analysis. Indeed, our experimental design suggests that neurofeedback training is responsible for the observed differences in CNV changes and that these changes are modulated by impulsivity. However, it does not shed light on specific or unspecific effects of the neurofeedback training, which are discussed actively in the neurofeedback field (Kober et al., 2017; Strehl et al., 2017; Witte et al., 2018) and need to be examined in future studies.

In addition, we expect that our model estimates are quite fragile and uncertain due to the small number of participants and the low signal-to-noise ratio of EEG measures. Using more informative priors or pooling data/resources from different studies sharing the same goal might decrease the associated epistemic and model uncertainty and provide the basis for stronger conclusions. Further investigations regarding changes in CNV in individuals with ASD throughout adolescence, along with tests of executive functions, would be necessary to understand the developmental trajectory and clinical significance of the CNV in ASD symptomatology.

## Conclusion

In summary, our study demonstrates that improvements in executive functions after SCP neurofeedback training, as indicated by the increase in the CNV following intervention, is limited to the group of young patients with deficits in impulsivity control. Future research is necessary to investigate whether the transdiagnostic subtypes suggested by the Research Domain Criteria Initiative are linked to different functional and neuroanatomical networks. In this way, impulsivity would not only be recognized as an underlying construct for several disorders in ICD or DSM (Moeller et al., 2001), but also its transdiagnostic neuropsychological mechanisms and subtle impact across mental disorders would be put in the spotlight.

## Data Availability Statement

The raw data supporting the conclusions of this article will be made available by the authors, without undue reservation.

## Ethics Statement

The studies involving human participants were reviewed and approved by the Ethics Committee of the Medical University of Vienna. Written informed consent to participate in this study was provided by the participants’ legal guardian/next of kin.

## Author Contributions

LK and LP conceived and designed the study. KP, SR, and KD conducted the measurements. SR, KP, and LK analyzed and interpreted the data. KP, SR, KD, LP and LK wrote, edited, and revised the manuscript. All authors contributed to the preparation of the manuscript and approved the final manuscript.

## Conflict of Interest

The authors declare that the research was conducted in the absence of any commercial or financial relationships that could be construed as a potential conflict of interest.

## Publisher’s Note

All claims expressed in this article are solely those of the authors and do not necessarily represent those of their affiliated organizations, or those of the publisher, the editors and the reviewers. Any product that may be evaluated in this article, or claim that may be made by its manufacturer, is not guaranteed or endorsed by the publisher.

## Footnotes

1 The same ensuing analysis was also performed following the *autoreject* algorithm (Jas et al., 2017) which determines an adaptive threshold for each data set. This analysis did not lead to substantially different results, so it was only considered as a robustness check.
